# High intensity interval running enhances measures of physical fitness but not metabolic measures of cardiovascular disease risk in healthy adolescents

**DOI:** 10.1186/1471-2458-13-498

**Published:** 2013-05-24

**Authors:** Duncan S Buchan, Stewart Ollis, John D Young, Stephen-Mark Cooper, Julian PH Shield, Julien S Baker

**Affiliations:** 1 Institute for Clinical Exercise and Health Science, School of Science, University of the West of Scotland, Hamilton, Scotland ML3 0JB, UK; 2 Cardiff School of Sport, Cardiff Metropolitan University, Cyncoed Campus, Cyncoed Road, Cardiff, UK; 3 School of Clinical Sciences, University of Bristol and Bristol Royal Hospital for Children, Bristol BS2 8AE, UK

## Abstract

**Background:**

With accumulating evidence suggesting that CVD has its origins in childhood, the purpose of this study was to examine whether a high intensity training (HIT) intervention could enhance the CVD risk profile of secondary school aged adolescents in a time efficient manner.

**Methods:**

Participants in the study were adolescent school children (64 boys, 25 girls, 16.7 ± 0.6 years). The intervention group (30 boys, 12 girls) performed three weekly exercise sessions over 7 weeks with each session consisting of either four to six repeats of maximal sprint running within a 20 m area with 30 s recovery. The control group were instructed to continue their normal behaviour. All participants had indices of obesity, blood pressure and nine biochemical risk markers for cardiovascular disease recorded as well as four physical performance measures at baseline and post-intervention. Feedback was provided through informal discussion throughout the intervention period as well as post-intervention focus groups. Statistical differences between and within groups were determined by use of paired samples t-tests and ANCOVA.

**Results:**

Significant enhancements (P ≤ 0.05) in vertical jump performance, 10 m sprint speed and cardiorespiratory fitness was evident in the intervention group whereas a significant decrease in both agility and vertical jump performance was evident in the control group. Participants in the intervention group also experienced a significant decrease in systolic blood pressure post-intervention. Limited changes occurred with respect to the biochemical markers although both groups did experience a significant increase in LDL post-intervention whilst the control group experienced a significant decrease in total cholesterol. No apparent differences were evident between groups post intervention for any of the biochemical markers. Feedback indicated that participants endorsed the use of the intervention as an effective means of exercise.

**Conclusions:**

Our results demonstrate that high intensity exercise interventions may be used in the school setting for adolescents as a means of improving measures of physical fitness. Further investigations involving a larger cohort of participants, taken from different schools, is recommended.

**Trial registration:**

NCT01027156

## Background

Atherosclerotic cardiovascular disease (CVD) is the leading cause of death in the Western world [1]. Whilst atherosclerosis is a lifetime disease, very few symptoms are evident until late into its course. The incidence of CVD results from a complex interaction of many risk factors, some of which are evident in youth [2-5], often while individuals are asymptomatic and unaware of the consequences. Childhood obesity, poor cardiorespiratory fitness (CRF) and physical inactivity are all independent risk factors for CVD disease but are also associated with other risk factors such as high blood pressure [5], type 2 diabetes, insulin resistance, adiponectin [6], C-reactive protein [4] and arterial stiffness [7]. Unsurprisingly, increasing physical activity levels is often purported as an important constituent of a healthy lifestyle given its beneficial effects upon both weight status and CRF.

Despite the well-established benefits of regular physical activity on health and well-being, current levels within school aged youth are widely regarded as insufficient to meet recommendations [8]. Recommendations within the United Kingdom recommend that all children and young people should engage in moderate to vigorous intensity physical activity for at least 60 minutes and up to several hours every day whilst also suggesting that vigorous intensity activities, including those that strengthen muscle and bone, should be incorporated at least three days a week [9]. Understanding why youth fail to meet recommended physical activity levels is a complex phenomenon influenced by numerous interrelated factors that can be different for each individual. Nonetheless, school is one setting where youth spend a substantial amount of their time. The school environment, and in particular physical education (PE), affords an ideal setting to practice health-promoting behaviours and is widely recognized as an important setting for collaborative intervention [10]. Moreover, the school setting provides a venue whereby interventions can reach a large number of individuals from assorted socio-economic surroundings while providing an environment for youth to engage in physical activity.

Even so, it is clear that many youth have difficulty, and perhaps little interest, in partaking in long duration endurance based activities. Of relevance may be the fact that many of the activities of youth are intermittent, and are of a high intensity [11] and are in contrast to the continuous nature of recommended and prescribed programmes. Recently, a growing body of evidence has found that the adaptations typically associated with traditional endurance exercise may also occur through low volume, high-intensity interval training (HIT) [12-16]. For example, our group have previously demonstrated significant reductions in systolic blood pressure (BP) and CRF in adolescents after 7 weeks of an HIT intervention [16]. Others utilizing different protocols have also demonstrated a significant increase in skeletal muscle markers of carbohydrate metabolism, lipid oxidation and mitochondrial biogenesis [12], CRF [14], increased endothelial function and CRF [13] and increased insulin action [15]. From these findings, some have suggested that HIT may provide a useful alternative to traditional endurance based exercise recommendations for health promotion. Particularly when you consider that lack of time is often cited as a common determinant of exercise participation regardless of sex, age, ethnicity or health status [17].

Nonetheless, the use of HIT interventions is often perceived as unpractical and intolerable by the general population. It is important therefore that further work is undertaken to support the role of alternative evidence based exercise recommendations. Thus, the purpose of this study was to examine the effects of a HIT intervention upon a number of risk factors of CVD. The working hypothesis of the present study was that HIT interventions are effective for the primary prevention of lifestyle-related disease risk factors.

## Methods

### Ethics statement

The study protocol was approved by the University of the West of Scotland Ethics committee and conformed to the Declaration of Helsinki.

The design, intervention and measurement protocols have been described in detail previously [18,19]. A short overview is provided below.

### Participants

A cohort of adolescent school children (64 boys, 25 girls, 16.7 ± 0.6 years) volunteered for the study. Informed consent was obtained for all volunteers and their parents. Participants were recruited from year 5 and year 6 PE classes. The total number of pupils in the two school years (5 and 6) was 236. However, only participants who chose to study PE (n = 92) were eligible. Of this, 89 agreed to participate providing a 97% recruitment rate. No other inclusion or exclusion criterion was applied. A quasi-experimental research design was utilized and the two year groups were then randomly assigned as either the high intensity training (HIT) or the control group. Participants were instructed not to change their dietary or lifestyle habits other than prescribed throughout the intervention period. Prior to experimental data collection all participants were fully habituated to experimental data collection and test procedures.

### Study design

An ecological model and action research/dynamic assessment methodology was used in this study. The basic premise of ecological models is to support individuals undertaking health enhancing behaviour by ensuring that the environments and policies, relevant to an individual, support such an approach [20,21]. In this study, we focused our efforts on the individual participant as well as the PE teachers and school management by forming collaborative partnerships with the research team. Forming collaborative partnerships is highly compatible with multilevel, multi-sectorial ecological models as they allow for on-going engagement and interaction, increasing the relevance and feasibility of initiatives [22]. This partnership based approach afforded the opportunity for two-way dialogue between all parties which fostered the collaborative nature of the intervention.

By utilizing an action research/dynamic assessment methodology, the team were able to gather substantial data and information concerning the intervention and adapt accordingly in an on-going basis, indicative of action research [23]. Regular communications of findings, progress and development were continually shared between all relevant parties and in doing so, informed future action points, strategies and plans throughout the intervention. The dynamic assessment component of our methodological approach was achieved through the use of several different approaches to evaluate the effectiveness of the intervention. This included weekly heart rate monitoring, weekly video monitoring, discussion groups and continued observation and feedback to all relevant parties concerning individual and group participation and responsiveness throughout the intervention. This occurred during and after each exercise session throughout the intervention period. By utilizing such an approach we were able to draw conclusions about the participants learning responses to the intervention which then informed future action points, strategies and plans throughout.

### Intervention

Participants in the control group (34 boys, 13 girls) were instructed to continue normal behaviour. Participants in the HIT group (30 boys, 12 girls) were required to complete a 30 s maximal effort sprint within a 20 m distance separated by cones. Validity and reliability of the 20 m sprint as a measure of anaerobic performance has been provided previously [24]. Initially, participants repeated the protocol four times with a 30 s recovery period between sprints which equated to 2 mins of maximal effort sprinting interspersed with 2 min recovery. The protocol was performed 3 times weekly. Training progression was implemented by increasing the number of repetitions from four during weeks 1 and 2, to five during weeks 3 and 4, to six during weeks 5 and 6. During week 7, participants still performed six repetitions but each was interspersed by only 20 s recovery. Overall, participants undertook 54 mins of exercise during the intervention period. Participants were given a familiarization trial of four low intensity runs prior to the start of the intervention. Timing of the sprint and the recovery period was recorded manually by the same investigator.

### Physical and physiological measures

Barefoot stature was measured to the nearest 1 mm (Seca Stadiometer, Seca Ltd, Birmingham, UK). Weight in normal PE clothing was measured to the nearest 0.1 kg using calibrated electronic weighing scales (Seca 880, Digital Scales, Seca Ltd, Birmingham, UK). Sexual maturation status was determined using a self-report questionnaire based on the criteria of Tanner [25] for stage of pubic hair (PH) development. Systolic BP and diastolic BP was measured with an automated monitor (Omron M10-IT Blood Pressure Monitor HEM-7080IT-E, Omron Healthcare UK Ltd, Milton Keynes, UK) after each participant had sat quietly for a period of 10 min. The average of the second and third measures was used for analysis.

Four components of health-related physical fitness was measured and included: CRF, muscular power and speed/agility. CRF was measured using the 20 m multi stage fitness test (20MSFT) which is viewed as a valid predictor of maximal CRF in young people [26]. The counter movement jump (CMJ) was used to measure muscular power with jumping height measured using the Optojump system (Microgate, Bolzano, Italy) after a standardized warm-up. Sprint (running) speed was measured over 10 m using an electronic sprint timer with photoelectric sensors (Polifemo Radio Light – Microgate, Italy). The 10 m sprint took place with photoelectric sensors placed at 0 and 10 m intervals. Agility was measured with the 505 test. Two photoelectric sensors (Polifemo Radio Light – Microgate, Italy) were placed 5 m from the starting line and 5 m from a designated turning point. Participants were instructed to sprint maximally to the designated turning point, pivot, and return as quickly as possible through the photoelectric sensors in accordance with standard procedures [27]. All measurements were taken at baseline and post-intervention in both groups at the same time of day in the school sports hall.

Heart rate (HR) response was recorded on all participants once during each training week with continuous heart rate telemetry (Hosand, TM200, Hosand Technology, Verbania, Italy). On completion of the activity session participants were provided with personalized feedback and were encouraged to ask questions. It was expected that personalized feedback would facilitate engagement and adherence with the intervention and aims of the study.

### Lifestyle questionnaires

All participants completed a validated physical activity questionnaire for adolescents (PAQ-A) [28] which required them to recall their physical activity behaviour from the previous 7 days. The questionnaire provides a score for each individual ranging from 0–5, with 0 reflecting no physical activity and 5 being very physically active. This measurement is particularly useful as an indicator of general activity patterns that can discriminate between active and inactive individuals rather than trying to estimate the intensity and duration of activities. Daily food intake was estimated with a validated, self-reported seven-day food diary [29] and a food frequency questionnaire. Participants were instructed to complete the food diary and record everything that they ate and drank over a specified seven-day period. Returned food diaries and questionnaires were inspected and when necessary, clarification of responses were confirmed with participants. The collated data were analyzed using the nutritional analysis software by Health Options Ltd (Nutri Check, Health Options Ltd, Cirencester, Gloucester, UK) with average daily kilocalories (kcal/d), percentage of total fat, and saturated fat then calculated.

### Metabolic measures

Blood samples were obtained between 9:00 am and 11:00 am after an overnight fast in all participants. Prior to sampling, participants were instructed to sit quietly for a period of at least 30 mins to control for plasma volume shifts [30]. A team of qualified phlebotomists, experienced in paediatric sampling techniques collected the blood samples. Blood samples were obtained from an antecubital vein and collected in a BD Vacutainer plasma tube (Becton, Dickinson and Company, Franklin Lakes, USA). Plasma was isolated by centrifugation at 3,500 rpm for 10 minutes and frozen at −80°C within two hours of collection. Analyses were subsequently completed within three months of collection. Total cholesterol, insulin, high-density lipoprotein cholesterol (HDL), low-density lipoprotein cholesterol (LDL), high-sensitivity C-reactive protein (CRP), glucose, interleukin-6 (IL-6), adiponectin and triglycerides were measured. All analyses were performed using standard procedures. Total cholesterol and triglycerides were measured by enzymatic methods (TR210 and CH200 Randox, Co. Antrim, UK) and a Camspec M107 spectrophotometer (Camspec, Leeds, UK). Concentration of HDL was determined after precipitation of very low density and low-density lipoproteins by the addition of phosphotungstic acid in the presence of magnesium ions. The Friedewald formula [31] was used to calculate LDL-C concentration. Glucose was measured using the glucose oxidase method (GL364, Randox, Co. Antrim, UK) and analyzed with a Camspec M107 spectrophotometer (Camspec, Leeds, UK). Plasma insulin was analyzed with commercially available immunoassay kits (ALPCO, Salem, NH, USA) and a Camspec M107 spectrophotometer (Camspec, Leeds, UK). Concentrations of IL-6, and CRP were measured with specific enzyme linked immune-sorbent assay (ELISA) kits (R & D Systems, Abingdon, UK) and a MRX microplate reader (Dynatech Laboratories, Cambridge, MA, USA). The inter (%) and intra (%) co-efficient of variance for the metabolic measures were as follows: IL-6 (2%, 8%), insulin (4%, 9%), adiponectin (11%, 10%), CRP (6%, 12%), glucose (7%, 4%), total cholesterol (4%, 3%), HDL (6%, 2%) and triglycerides (5%, 6%).

### Observational analysis

Observational data was gathered by video recording. One weekly exercise session was recorded which permitted the estimation of distances covered by the HIT group during the week.

### Intervention feedback

The feasibility of the intervention was evaluated through five focus groups: four involved the exercise participants (n=24) and one involved the PE teachers (n=4). The focus groups were facilitated by two members of the research team who had been in attendance throughout the study. One member of the research team acted as the moderator and was responsible for facilitating the group discussion. The second member of the research team acted as the co-moderator and was responsible for taking notes during the sessions. After each focus group, the moderator and co-moderator debriefed and described any noticeable circumstances that influenced discussions. Focus groups were audio recorded and transcribed after which a discourse analysis was performed. By utilizing this method, the transcripts of interviews were searched for unique information, common patterns and uncommon patterns [32]. Integrated reliability was achieved through line by separate line coding of the transcripts by the moderator and co-moderator. Themes were identified by each and then compared to identify final themes. Reviewing the transcripts allowed for identification of common themes within single, and across focus groups. Representative quotes from each identified thematic area are provided. Focus groups ranged in length from 30 minutes to one hour. Throughout the intervention period, informal discussions were also undertaken with the PE teachers, members of school management and participants either individually or in small groups. These discussions were frequent and allowed us to gain immediate feedback of the intervention.

### Statistical analysis

A statistical power of 0.8 and a significance level of 0.05 was used throughout the study. In order to determine sensitivity to change and the effects of the intervention upon CVD risk factors, sample size was estimated using the procedures of Park and Schutz [33] for ANOVA designs that incorporate a repeated factor. For a medium effect size in the intervention groups of d = 0.5, power of 0.8 (suggesting an 80% probability of achieving significance at the p = 0.05 level) a sample group size of 17 participants was required. Effect sizes > 0.5 indicate clinically relevant changes [34]. Assuming a drop-out rate of 10%, a minimum of 19 participants was needed per group. Means (±SD) were used to describe data where appropriate.

The paired samples t-test was used to establish any differences within pre and post measures for each group after the Anderson–Darling test was applied to confirm normality and Levene’s test assessed homogeneity of variance. For variables with invalid assumptions of normality and (or) homogeneity of variance, the Wilcoxon signed rank test was used.

Additionally, the analysis of covariance (ANCOVA) was used to test differences between the groups at post-intervention. The covariates (baseline age, sex, maturation, physical activity, total dietary fat, saturated fat and kcal/d difference as well as differences in waist circumference and shuttle run score from baseline to post-intervention) were used in the model based on the results from two exploratory analyses: i) confirmation that the potential covariate was independent of experimental effects by establishing that the two groups did not differ significantly on each of these variables via the one-way analysis of variance (ANOVA), and, ii) that each of the potential covariates correlated significantly with each of the dependent variables. The underlying assumptions of residual normality and homogeneity of variance were confirmed by the Anderson-Darling test and Levene’s test respectively for all measures. Finally, post hoc, effect size statistics (ES) for all the statistically significant *t*-ratios and *F*-ratios identified, as well as the 95% confidence intervals (CI) for the mean differences for each group was presented. Only participants that had complete data (i.e. both baseline and post-intervention measures) were included in the analysis for each variable.

## Results

Overall, 20 participants were classified as overweight with 69 being of a healthy weight [35]. Table 1 displays the baseline characteristics for both groups. Mean PA levels estimated with the PAQ-A was similar between groups suggesting that participants were not overtly active. Dietary habits were also similar with both groups consuming diets greater than the recommended threshold for saturated and total fat intake [36]. Of the 21 exercise sessions, mean attendance for participants involved in the intervention was 16.6 ± 1.5. Absences were due to illness, medical appointments, lack of appropriate clothing or work placement attendance. No injuries were reported during the intervention and in terms of compliance; no participants withdrew from the study. This is encouraging, since intense vigorous exercise in youth is often dismissed as being unfeasible for many to undertake. Weekly HR response and distance covered during one session per week are displayed in Table 2. Mean HR responses throughout the intervention was comparable with HIT and for recommendations aimed at improving CRF [37].

**Table 1:** Mean age, maturity, physical activity and dietary characteristics of both groups at baseline

	**HIT group (n = 42)**	**Control (n = 47)**
Age	16.8 ± 0.5	16.6 ± 0.6
Physical activity levels	2.3 ± 0.4	2.1 ± 0.7 (44)*
**Tanner stage**		
Pre-pubescents (Stage 1)	5	1
Pubescents (Stages 2, 3 and 4)	25	21
Post-pubescents (Stage 5)	12	25
**Dietary measures**		
Total fat	36.4 ± 4.5 (38)*	36.1 ± 4.6 (41)*
Saturated fat	13.6 ± 2.2 (38)*	13.7 ± 2.2 (41)*
Total calories	1723.8 ± 469.4 (38)*	1569.2 ± 365.1 (41)*

*Where n = denoted number, actual sample number is presented in.

**Table 2:** Average heart rate response and distance covered (mean ± SD) during each week of the intervention

	**HIT group (n = 42)**
**Average weekly distance covered (m)**	
Week 1	495.5 ± 45.1 (39)*
Week 2	496.8 ± 44.2 (37) *
Week 3	617.7 ± 55.6 (35) *
Week 4	621.7 ± 60.1 (36) *
Week 5	761.1 ± 32.7 (34) *
Week 6	762.7 ± 39.6 (35) *
Week 7	746.1 ± 41.6 (36) *
**Average weekly heart rate response (bpm)**	
Week 1	175.4 ± 15.6 (32) *
Week 2	172.6 ± 14.2 (30) *
Week 3	175.5 ± 12.6 (27) *
Week 4	178.3 ± 16.7 (26) *
Week 5	181.3 ± 15.9 (28) *
Week 6	178.4 ± 18.7 (27) *
Week 7	176.4 ± 19.3 (26) *

Note: * Where n = denoted number, actual sample number is presented in brackets.

### Physical and physiological variables post-intervention

Table 3 displays the mean ± SD for physical and physiological measures for the HIT and control groups at baseline and post-intervention as well as the differences between, and within, the groups post-intervention. Both groups experienced a significant increase in stature whilst only the control group displayed significant increases in mass and waist circumference. Participants in the HIT group also experienced a significant decrease in systolic BP post-intervention. There was a significant decrease in both agility and vertical jump performance in the control group whereas increases in vertical jump, 10 m sprint speed and CRF performance was evident in the HIT group. Positive changes in agility performance followed the HIT intervention but did not reach significance. A negative change in CRF was evident in the control group post-intervention but again, this did not reach significance.

**Table 3:** Characteristics of physical and physiological variables: HIT and control group at baseline and post-intervention

**Characteristic**	**Baseline**	**Post-intervention**	**Mean difference**	**Effect size**	**Within group**	**Between groups**
			**(95%CI)**	**(Power)**	**P- value**	**P-value**
						**(Post Intervention)**
Stature (cm)						0.821
HIT (42)	170.2 ± 8.3	171.1 ± 8.3	0.9 (0.6 to 1.3)	0.76 (95%)	0.000	
Control (46)	171.6 ± 8.2	172.5 ± 8.2	0.9 (0.6 to 1.2)	0.87 (95%)	0.000	
Mass (kg)						0.918
HIT (38)	70.4 ± 8.9	70.2 ± 9.0	0.2 (-0.8 to 0.3)		0.350	
Control (47)	71.9 ± 9.7	72.4 ± 9.4	0.5 (0.3 to 0.9)	0.32 (56%)	0.036	
BMI					0.168	0.842
HIT (38)	21.5 ± 2.4	21.3 ± 2.3	0.2 (0.1 to 0.5		0.178	
Control (46)	22.6 ± 2.6	22.3 ± 2.5	0.3 (-0.2 to 0.4)			
Waist circumference (cm)						0.695
HIT (42)	75.3 ± 6.7	75.3 ± 6.7	0.002 (-0.7 to 0.7)		0.995	
Control (47)	73.9 ± 5.9	75.4 ± 6.2	1.5 (1.0 to 2.0)	0.91 (99.9%)	0.000	
Systolic BP (mmHg)						0.146
HIT (38)	119 ± 13	114 ± 13	4.9 (-8.4 to -1.4)	0.46 (78.6%)	0.008	
Control (39)	119 ± 14	116 ± 15	3.3 (-7.4 to 0.8)		0.109	
Diastolic BP (mmHg)						0.672
HIT (38)	69 ± 11	68 ± 9	0.9 (-5.5 to 3.7)		0.689	
Control (39)	70 ± 12	66 ± 12	3.5 (-8.3 to 1.3)		0.144	
CMJ (cm)						0.042
HIT (41)	30.7 ± 6.7	31.7 ± 7.1	1.0 (0.3 to 1.6)	0.49 (86.6%)	0.003	
Control (38)	31.8 ± 6.6	29.7 ± 6.3	2.1 (-3.0 to -1.1)	0.72 (99.1%)	0.000	
10 m sprint (s)						0.003
HIT (37)	2.06 ± 0.26	1.97 ± 0.21	0.09 (-0.12 to -0.05)	0.78 (99.6%)	0.000	
Control (37)	2.04 ± 0.20	2.01 ± 0.18	0.03 (-0.01 to 0.06)		0.167	
5-0-5 agility (s)						0.095
HIT (42)	2.58 ± 0.28	2.54 ± 0.30	0.05 (-0.10 to 0.01)		0.090	
Control (37)	2.45 ± 0.25	2.58 ± 0.28	0.13 (-0.18 to -0.07)	0.79 (99.7%)	0.000	
CRF (Shuttles)						0.277
HIT (41)	79 ± 25	84 ± 26	4.6 (2.8 to 6.5)	0.78 (99.8%)	0.000	
Control (47)	82 ± 25	78 ± 22	3.4 (-6.9 to 0.1)		0.056	

Abbreviations: *HIT* High Intensity group, *BP* Blood Pressure, *CRF* CRF, *CMJ* Counter movement jump, *CI* confidence interval. Paired samples t-test was used to establish differences between post-intervention measures for each group. ANCOVA was used to test differences between the groups at post-intervention adjusted for the covariates (baseline age, sex, maturation, physical activity, total dietary fat, saturated fat and kcal/d difference as well as differences in waist circumference and shuttle run score) where appropriate.

Finally, results from the ANCOVA established the differences between the groups post-intervention (Table 3). There was a significant difference in vertical jump (P = 0.042, ES = 0.54, power = 62.6%) and 10m sprint (P = 0.003, ES = 0.85, power = 93.1%) performance after partitioning-out baseline measures of sex, maturation, physical activity and kcal/d.

### Biochemical variables for each group at baseline and post-intervention

Table 4 displays the mean ± SD of the biochemical variables for the HIT and control groups at baseline and post-intervention as well as the differences between, and within the groups post-intervention. Both groups experienced a significant increase in LDL post-intervention whereas the control group also displayed a significant increase in total cholesterol levels. No other significant changes were evident in either group post-intervention for the other biochemical CVD risk factors measured. Finally, results from the ANCOVA revealed no significant differences between the groups post-intervention for any measure.

**Table 4:** Biochemical characteristics: HIT and control group at baseline and post-intervention

**Characteristic**	**Baseline**	**Post-intervention**	**Mean difference**	**Effect size**	**Within group**	**Between groups P-value**
			**(95%CI)**	**(Power)**	**P- value**	**(Post-intervention)**
Adiponectin (ng ml^-1^)						0.985
HIT (37)	9.4 ± 6.1	7.6 ± 6.5	1.8 (-4.4 to 0.8)		0.162	
Control (46)	8.1 ± 4.5	7.4 ± 6.2	0.6 (-2.3 to 1.0)		0.191	
CRP (mg L^-1^)						0.940
HIT (36	1.3 ± 1.2	1.6 ± 1.7	0.4 (-0.2 to 1.1)		0.192	
Control (43)	1.4 ± 1.4	1.6 ± 1.6	0.2 (-0.4 to 0.8)		0.459	
Glucose (mMol L^-1^)						0.517
HIT (26)	4.9 ± 1.3	5.0 ± 1.2	0.1 (-0.6 to 0.7)		0.836	
Control (38)	5.0 ± 1.0	5.2 ± 1.0	0.2 (-0.2 to 0.6)		0.434	
HDL (mMol L^-1^)						0.694
HIT (26)	1.4 ± 0.3	1.8 ± 1.1	0.3 (-0.1 to 0.7)		0.167	
Control (36)	1.4 ± 0.4	1.7 ± 1.0	0.4 (-0.01 to 0.8)		0.056	
Insulin (IU ml^-1^)						0.235
HIT (24)	7.9 ± 7.5	8.5 ± 11.8	0.6 (-5.5 to 6.7)		0.764	
Control (34)	7.3 ± 4.5	5.8 ± 4.2	1.5 (-3.6 to 0.6)		0.307	
IL-6 (pg ml^-1^)						0.495
HIT (27)	3.4 ± 4.1	2.9 ± 3.8	0.6 (-2.1 to 0.9)		0.788	
Control (40)	3.8 ± 2.9	3.9 ± 7.9	0.2 (-2.6 to 2.9)		0.149	
LDL (mMol L^-1^)						0.402
HIT (24)	2.5 ± 1.5	1.5 ± 1.0	1.1 (-2.0 to -0.2)	0.51 (67.6%)	0.019	
Control (33)	2.8 ± 1.8	1.7 ± 0.8	0.9 (-1.6 to -0.3)	0.51 (80.8%)	0.006	
Total Cholesterol (mMol L^-1^)						0.182
HIT (28)	4.5 ± 1.8	3.8 ± 1.2	0.7 (-1.6 to 0.2)		0.149	
Control (40)	4.2 ± 1.6	3.5 ± 1.3	0.7 (-1.5 to -0.05)	0.34 (56.1%)	0.036	
Triglycerides (mMol L^-1^)						0.609
HIT (29)	1.0 ± 0.3	1.1 ± 0.4	0.1 (-0.1 to 0.3)		0.172	
Control (41)	1.0 ± 0.4	1.1 ± 0.5	0.05 (-0.1 to 0.2)		0.526	

Abbreviations: *HIT* High Intensity group, *CI* confidence interval, *CRP* C-reactive protein, *HDL* high-density lipoprotein cholesterol, *IL-6* interleukin-6, *LDL* low-density lipoprotein cholesterol. Paired samples t-test was used to establish differences between post-intervention measures for each group. ANCOVA was used to test differences between the groups at post-intervention adjusted for the covariates (baseline age, sex, maturation, physical activity, total dietary fat, saturated fat and kcal/d difference as well as differences in waist circumference and shuttle run score) where appropriate.

### Intervention feedback

#### Exercise participants

Intervention participants provided feedback concerning the intervention through post-intervention focus groups (Additional file 1). Two broad themes are highlighted below, which are grounded in the emergent categories from participant responses. Each theme is supported by quotations from participants.

### Theme one

#### Participant’s comments regarding the intervention

Participants reported that they enjoyed the vigorous nature of the intervention with one stating “We don’t usually do a lot of running about in PE so it was good to actually do something”. This sentiment was agreed by a number of individuals. A number of participants felt that the intervention whilst hard at first became gradually easier as the weeks progressed. One stated: “I thought there was no way I’m doing this for 7 weeks but once it (the exercise session) was finished, you feel really good” whilst another stated “At the start of a week when the reps increased I was dreading it but once you do a couple of sessions it becomes easy and you know what to expect”. This was an important point which was confirmed in each focus group. It became apparent that whilst participants had initial fears at the beginning, once they undertook the exercise they felt more confident about subsequent sessions.

### Theme two

#### Participants continued participation with the intervention

Participants identified a number of reasons for their continued participation. These included: “I just wanted to get fit “, “I didn’t want to let XXXXXX down as we have been doing it together since the start”, “I just wanted to make sure I was beating XXXX as we were racing each other”. Other participants reported: “We didn’t want to let anyone down as we knew there was a lot of money spent”. Another important point raised by a number of participants concerned the feedback and encouragement given. One stated ”It was good seeing the heart rates on the computer as we knew how hard we were running” whilst another stated “Feedback was good and everyone on the sides was cheering you on”. Three main reasons that were given for their continued participation of the intervention were participant support, competition and not letting anyone down. They also indicated that the support and encouragement given was important for continued participation.

### PE teachers

Teachers provided feedback concerning the intervention through a post-intervention focus group (Additional file 2). Four departmental staff members were in attendance. Two broad themes are highlighted below with each supported by quotations from participants.

### Theme one

#### Commenting upon the intervention

The teachers all agreed that they really enjoyed the experience of the intervention. One stated “It went really well. There was no way I thought the kids would continue to do it over the 7 weeks but the fact they did, was great. You could see as well that they really got into it.” While another stated “I was surprised how much I cared about the study, I know it’s your research but I felt that it was ours too. It was all we ever talked about in the mornings”. This comment was confirmed by all other teachers in attendance with another adding “Some of the kids really got into it and showed massive improvements, even those that you wouldn’t expect.” The teachers all agreed that the heart rate sessions were great; not only for the participants, but also for the staff with one commenting “It was great for the pupils to see their HR response during and after exercise.”

### Theme two

#### Participant’s adherence to the intervention

The teachers agreed that the positive influence of the researchers in attendance throughout the study was a key contributor to the successful adherence of participants. One commented “It was clear that you (the two researchers) were really engaged with the participants and were willing to provide support and encouragement to all. The relationships that you had with the kids were also appropriate; we were amazed that after only 1 week you knew the names of all the kids”. Teachers also noted that they bonded with the kids much earlier in the year than usual with one stating “I got to know the kids really well because we were all involved in this study. It was good.”

## Discussion

The aim of this study was to establish the effects of a HIT intervention upon a number of CVD risk factors in adolescent youth. Results revealed that body mass and waist circumference was maintained in the HIT group although significant increases in these measures were evident in the control group. This suggests that the intervention may have had an impact on body mass and waist circumference maintenance by limiting their increase during the intervention period as observed within the control group. Although previous investigations involving youth have noted significant reductions in body composition through a HIT intervention [13,14], both of these studies were of a longer duration involving overweight and obese participants. Given the short duration of this study and that only 2 individuals had at risk waist circumference levels (data not shown), our findings are unsurprising.

A significant reduction in systolic BP and increase in CRF post-intervention confirms our findings from an earlier investigation [16]. Reductions in systolic BP through aerobic exercise training are well established with approximate reductions of 6 to 10 mm Hg seen in previously sedentary men and women of all ages [38]. This decrease in systematic vascular resistance through aerobic exercise training has also been linked to the reduction in systolic BP [39] and appears to suggest that central adaptations may be responsible for the adaptations noted in the HIT group. Nonetheless, previous HIT investigations involving healthy adults appear to contradict the suggestion that central adaptations are linked to an increase in CRF [40,41].

For instance, participants involved in a 6 week HIT intervention undertaken 3 times per week experienced significant peripheral (a-vO_2_ difference) but not central (maximal cardiac output) adaptations [41] whereas in the study by Rakobowchuk and colleagues, an intervention undertaken 5 times per week for 6 weeks improved peripheral, but not central, vascular structure [40]. Whether peripheral adaptations can explain the increase in CRF evident in this cohort is unclear. Given the nature of our school based intervention it was not possible to undertake similar measures in this cohort given the number of participants and the time restrictions of the school curriculum. Nonetheless, previous investigations that have involved HIT interventions have consistently demonstrated peripheral adaptation through improved oxygen delivery to working muscles [41], enzymatic [42] and mitochondrial adaptations [12,42]. Furthermore, some have also suggested that reductions in systolic BP from aerobic exercise training are due to an increased stroke volume through improved systemic vascular resistance (peripheral adaptation) rather than an increased cardiac output [39]. This is plausible since resting HR typically decreases after a period of exercise. From these studies it is evident that HIT interventions can stimulate peripheral adaptations which can enhance CRF, and perhaps systolic BP, and may explain the adaptations noted in this cohort.

Although these adaptations may be surprising given the initial level of CRF evident in this cohort (mean baseline 20MSFT levels of the cohort was in the top 90th percentile [43]), the design of our protocol may have ensured a large aerobic contribution towards energy production. Whilst our understanding of exercise metabolism is limited by ethical and methodological constraints in youth populations, evidence from adults may offer a plausible explanation for the increase in CRF evident in the intervention group. For instance, at the onset of the first repetition maximal power generation will partly be reliant upon muscle PCr stores to contribute towards ATP regeneration. As each exercise repetition is of 30s duration interspersed with 30s recovery, the decrease in PCr availability during subsequent repetitions [44] means individuals will rely more heavily upon both glycolytic and in particular, oxidative phosphorylation for continued energy production [45]. It was evident in this study that participants were unable to cover the same distance during subsequent bouts of exercise, especially towards the end of the intervention period (data not shown), which could indicate a greater reliance upon oxidative phosphorylation as exercise continues. This reliance may have become more pronounced in the latter repetitions of each session and towards the end of the intervention period given the inclusion of more repetitions and reduced recovery period, and is supported by our observations of the distances covered. It may be that the 1:1 exercise and recovery ratio provides enough stimuli to induce aerobic adaptations within the mitochondria (peripheral adaptation) since it is required to replenish ATP at a high rate with a decreasing anaerobic contribution [46].

Significant improvements in CMJ and 10 m sprint performance were noted in the HIT group whereas the control group experienced a significant decrease in CMJ performance only. It appears that the intervention which involved maximal acceleration and deceleration phases as well as explosive turns had a positive effect on muscular power, and confirms the findings of others that have shown an increase in muscular power through HIT interventions [19,47]. A logical explanation is that neurological adaptations (motor-unit recruitment) may explain the increase noted in explosive muscular power but evidence of this in adolescents is scarce. Although changes in agility favoured the HIT group, this did not reach significance while the control group experienced a significant decrease in agility post-intervention. It is well established that an increase in agility performance and maximal sprinting speed are reliant upon adaptations in a number of factors including muscular power, acceleration speed, balance and coordination [48]. While it appears that the HIT intervention was appropriate to induce significant increases in acceleration speed and muscular power (Table 3), it may be that balance and coordination did not improve at a sufficient level to induce a significant increase in agility performance.

A significant decrease in LDL concentration was evident in both the HIT and control groups while total cholesterol also decreased significantly in the control group. The large decrease in LDL evident in both groups post-intervention (Table 4) would have a subsequent effect upon total cholesterol levels, and can explain the significant reduction noted within the control group. Others have found similar positive effects of exercise on LDL in adolescent cohorts [49,50] while others have not [51]. Although participants were encouraged to maintain normal physical activity and dietary behaviours throughout the intervention period, the decrease in LDL evident in the control group could have been a result of increased physical activity, improved diet or a combination of both despite our recommendations. This however doesn’t appear plausible given that both body mass and waist circumference significantly increased post-intervention in the control group whereas CMJ and 10m sprint performance significantly decreased, as did CRF and 10-m sprint performance (albeit not significantly). It’s more likely that the dietary and physical activity behaviour of those participants in the control group was poor throughout the intervention period.

No significant differences were evident in the seven other metabolic CVD risk factors measured post-intervention but is unsurprising given the apparent healthy nature of this adolescent cohort. For instance, mean baseline 20 MSFT levels for both groups were in the top 90th percentile [43] while only 2 individuals presented with at risk waist circumference (data not shown). It is plausible that individuals who have poor anthropometrical and CRF profiles would have unfavourable metabolic profiles. Perhaps these individuals may be more susceptible to positive metabolic changes through the HIT intervention.

The findings from this study indicate that it is feasible to implement HIT interventions for adolescents within the school setting and for them to adhere to such a rigorous protocol. Findings from the focus groups and informal discussions revealed that adolescents are able to undertake strenuous intensive exercise and enjoy the nature of the activity. Whilst enjoyment was not specifically measured, we are confident that participant responses are accurate given that the mean adherence rate for the 21 exercise sessions was 16.6 ± 1.5 (~80%).

An additional important result from this study was the role of the PE teachers. Despite their initial trepidation and concerns regarding the intervention, unbeknown to us, the PE teachers had coordinated their lunch break to perform the HIT intervention prior to the start of the study. From informal discussions, the teachers themselves wanted to undertake the intervention to experience the challenges the pupils would soon face. Once the teachers began to provide positive feedback of their own experience of the intervention, we agreed that they would take an active role in emphasizing the importance and benefits of completing the intervention to the participants. As our relationship with the teachers strengthened, we focused our attention towards building relationships with the participants. As we were present during each exercise session, we were able to engage in informal discussions with participants and understand their reasons for participating. At the very least, this gave us a starting point for discussion during the subsequent sessions and weeks and was viewed positively by the participants during the focus group sessions post-intervention. From our experience, the strong relationships built with both teachers and participants allowed us to solve any issue that arose together, and appears vital for maintaining adherence and facilitating research outcomes.

The findings of this study, as well as our previous investigations [16,19] suggest that HIT may be an effective strategy to enhance measures of physical fitness in adolescent cohorts. As our studies have used apparently healthy adolescent cohorts, it is unsurprising that limited changes have occurred in the metabolic profiles of cohorts post-intervention. Also, the observations of this study are limited as it was not possible for allocation concealment or blinding since this study was conducted in only one school. Whilst attempts to reduce this confounding bias were undertaken by controlling for confounding variables in our analysis, it is plausible that the experimental treatment effect of the HIT intervention may have been exaggerated in this study. So whilst our findings suggest that HIT is a feasible method of physical activity in adolescent cohorts, further work is now required to establish the effects of HIT interventions on measures of health and well-being in a larger cohort of school children, taken from different schools through the use of a clustered randomized controlled trial.

## Conclusions

Findings from this study, suggest that HIT is an effective strategy to enhance measures of physical fitness in adolescent cohorts. Nonetheless, questions remain concerning the role of HIT as an effective alternative for health and well-being over more traditional endurance based exercise recommendations. Our studies have used apparently healthy adolescent cohorts but limited evidence exists concerning the use of HIT in individuals who have, or in risk of, clinical disorders such as CVD, type 2 diabetes, insulin resistance and obesity. Furthermore, the majority of HIT interventions have only been conducted over a relatively short period of time (i.e. several weeks). Whether HIT interventions can be implemented over an extended period of time (i.e. months to years) is at present unclear. Whilst we hope that our findings will stimulate further research related to the role of field based HIT in a variety of different populations, attention should also be given to identifying the optimal combination of exercise intensity and volume needed to induce favourable adaptation in the most time efficient manner. Further work that considers these issues whilst extending our initial work in a larger cohort of school children, taken from different schools within a clustered randomized controlled trial, will help provide evidence based recommendations for a variety of populations that may confirm the use of HIT as an alternative physical activity strategy.

## Competing interests

The authors declare that they have no competing interests.

## Authors' contributions

DSB led the overall process, collected all of the measures and designed the study. SO assisted with the data collection. JDY coordinated and carried out the blood sample analysis. SMC participated in the design of the study and performed the statistical analysis. JPHS participated in the coordination of the study and helped to draft the manuscript. JSB assisted with the design of the study and helped to draft the manuscript. All authors read and approved the manuscript.

## Pre-publication history

The pre-publication history for this paper can be accessed here:

http://www.biomedcentral.com/1471-2458/13/498/prepub

## Supplementary Material

Additional file 1 Focus Group Script – Participants. Click here for file

Additional file 2 Focus Group Script – PE Teachers. Click here for file

## References

[B1] Lloyd-Jones D Adams RJ Brown TM Carnethon M Dai S De Simone G Ferguson TB Ford E Furie K Gillespie C Heart disease and stroke statistics–2010 update: a report from the American Heart Association Circulation 2009 121 7 e46 e215 20019324 10.1161/CIRCULATIONAHA.109.192667

[B2] Freedman DS Khan LK Dietz WH Srinivasan SR Berenson GS Relationship of childhood obesity to coronary heart disease risk factors in adulthood: the Bogalusa Heart Study Pediatrics 2001 108 3 712 718 10.1542/peds.108.3.712 11533341

[B3] Raitakari OT Juonala M Kahonen M Taittonen L Laitinen T Maki-Torkko N Jarvisalo MJ Uhari M Jokinen E Ronnemaa T Cardiovascular risk factors in childhood and carotid artery intima-media thickness in adulthood: the Cardiovascular Risk in Young Finns Study J A M A 2003 290 17 2277 2283 10.1001/jama.290.17.2277 14600186

[B4] Cook DG Mendall MA Whincup PH Carey IM Ballam L Morris JE Miller GJ Strachan DP C-reactive protein concentration in children: relationship to adiposity and other cardiovascular risk factors Atherosclerosis 2000 149 1 139 150 10.1016/S0021-9150(99)00312-3 10704625

[B5] Juhola J Magnussen CG Viikari JS Kahonen M Hutri-Kahonen N Jula A Lehtimaki T Akerblom HK Pietikainen M Laitinen T Tracking of serum lipid levels, blood pressure, and body mass index from childhood to adulthood: the Cardiovascular Risk in Young Finns Study J Pediatr 2011 159 4 584 590 10.1016/j.jpeds.2011.03.021 21514597

[B6] Punthakee Z Delvin EE O'Loughlin J Paradis G Levy E Platt RW Lambert M Adiponectin, adiposity, and insulin resistance in children and adolescents J Clin Endocrinol Metab 2006 91 6 2119 2125 10.1210/jc.2005-2346 16537675

[B7] Ferreira I van de Laar RJ Prins MH Twisk JW Stehouwer CD Carotid Stiffness in Young Adults: A Life-Course Analysis of its Early Determinants Hypertension 2012 59 1 54 61 10.1161/HYPERTENSIONAHA.110.156109 22068867

[B8] Ekelund U Tomkinson G Armstrong N What proportion of youth are physically active? Measurement issues, levels and recent time trends Br J Sports Med 2011 45 11 859 865 10.1136/bjsports-2011-090190 21836170

[B9] Chief Medical Officers of England S, Wales, and Northern Ireland Start Active, Stay Active. A report on physical activity for health from the four home countries’ Chief Medical Officer 2011 London: Department of Health

[B10] Dobbins M De Corby K Robeson P Husson H Tirilis D School-based physical activity programs for promoting physical activity and fitness in children and adolescents aged 6–18 Cochrane Database Syst Rev 2009 1 CD007651 10.1002/14651858.CD007651 19160341

[B11] Strong WB Malina RM Blimkie CJ Daniels SR Dishman RK Gutin B Hergenroeder AC Must A Nixon PA Pivarnik JM Evidence based physical activity for school-age youth J Pediatr 2005 146 6 732 737 10.1016/j.jpeds.2005.01.055 15973308

[B12] Burgomaster KA Howarth KR Phillips SM Rakobowchuk M Macdonald MJ McGee SL Gibala MJ Similar metabolic adaptations during exercise after low volume sprint interval and traditional endurance training in humans J Physiol 2008 586 1 151 160 17991697 10.1113/jphysiol.2007.142109 PMC2375551

[B13] Tjonna AE Stolen TO Bye A Volden M Slordahl SA Odegard R Skogvoll E Wisloff U Aerobic interval training reduces cardiovascular risk factors more than a multitreatment approach in overweight adolescents Clin Sci 2009 116 4 317 326 10.1042/CS20080249 18673303

[B14] Gutin B Barbeau P Owens S Lemmon CR Bauman M Allison J Kang HS Litaker MS Effects of exercise intensity on cardiovascular fitness, total body composition, and visceral adiposity of obese adolescents Am J Clin Nutr 2002 75 5 818 826 11976154 10.1093/ajcn/75.5.818

[B15] Babraj JA Vollaard NB Keast C Guppy FM Cottrell G Timmons JA Extremely short duration high intensity interval training substantially improves insulin action in young healthy males BMC Endocr Disord 2009 9 3 10.1186/1472-6823-9-3 19175906 PMC2640399

[B16] Buchan DS Ollis S Young JD Thomas NE Cooper SM Tong TK Nie J Malina RM Baker JS The effects of time and intensity of exercise on novel and established markers of CVD in adolescent youth Am J Hum Biol 2011 23 4 517 526 10.1002/ajhb.21166 21465614

[B17] Godin G Desharnais R Valois R Lepage L Jobin J Bradet R Differences in perceived barriers to exercise between high and low intenders: observations among different populations Am J Health Promot 1994 8 4 279 285 10.4278/0890-1171-8.4.279

[B18] Buchan DS Ollis S Thomas NE Baker JS The influence of a high intensity physical activity intervention on a selection of health related outcomes: an ecological approach BMC Publ Health 2010 10 1 8 10.1186/1471-2458-10-8 PMC2830172 20064208

[B19] Buchan DS Ollis S Thomas NE Buchanan N Cooper SM Malina RM Baker JS Physical activity interventions: effects of duration and intensity Scand J Med Sci Sports 2011 21 6 e341 e350 10.1111/j.1600-0838.2011.01303.x 21518010

[B20] Stokols D Allen J Bellingham RL The social ecology of health promotion: implications for research and practice Am J Health Promot 1996 10 4 247 251 10.4278/0890-1171-10.4.247 10159704

[B21] King AC Stokols D Talen E Brassington GS Killingsworth R Theoretical approaches to the promotion of physical activity: forging a transdisciplinary paradigm Am J Prev Med 2002 23 2 Suppl 15 25 12133734 10.1016/s0749-3797(02)00470-1

[B22] Gillies P Effectiveness of Alliances and Partnerships for Health Promotion Health Promot Int 1998 13 2 99 120 10.1093/heapro/13.2.99

[B23] Baum F MacDougall C Smith D Participatory action research J Epidemiol Community Health 2006 60 10 854 857 10.1136/jech.2004.028662 16973531 PMC2566051

[B24] Baker J Ramsbottom R Hazeldine R Maximal shuttle running over 40 m as a measure of anaerobic performance Br J Sports Med 1993 27 4 228 232 10.1136/bjsm.27.4.228 8130958 PMC1332009

[B25] Tanner JM Whitehouse RH Clinical longitudinal standards for height, weight, height velocity, weight velocity, and stages of puberty Arch Dis Child 1976 51 3 170 179 10.1136/adc.51.3.170 952550 PMC1545912

[B26] Leger LA Mercier D Gadoury C Lambert J The multistage 20 metre shuttle run test for aerobic fitness J Sports Sci 1988 6 2 93 101 10.1080/02640418808729800 3184250

[B27] Draper JA Lancaster MG The 505 test: a test for agility in the horizontal plane Aust J Sci Med Sport 1985 17 1 15 18

[B28] Kowalski KC Crocker PRE Kowalski NP Convergent validity of the physical activity questionnaire for adolescents Pediatr Exerc Sci 1997 9 342 352

[B29] Food Standards Agency McCance and Widdowson’s the Composition of Foods 2002 6 Cambridge UK: Royal Society of Chemistry 29 379

[B30] Pronk NP Short term effects of exercise on plasma lipids and lipoproteins in humans Sports Med 1993 16 6 431 448 10.2165/00007256-199316060-00006 8303142

[B31] Friedewald WT Levy RI Fredrickson DS Estimation of the concentration of low-density lipoprotein cholesterol in plasma, without use of the preparative ultracentrifuge Clin Chem 1972 18 6 499 502 4337382

[B32] Kirby SL Greaves L Reid C Research Social Change: Methods Beyond the Mainstream 2006 2 Ontario: Broadview Press 219 254

[B33] Park I Schutz RW An introduction to latent growth models: analysis of repeated measures physical performance data Res Q Exerc Sport 2005 76 2 176 192 10.1080/02701367.2005.10599279 16128485

[B34] Cohen J A power primer Psychol Bull 1992 112 1 155 159 19565683 10.1037//0033-2909.112.1.155

[B35] Cole TJ Bellizzi MC Flegal KM Dietz WH Establishing a standard definition for child overweight and obesity worldwide: international survey BMJ 2000 320 7244 1240 1243 10.1136/bmj.320.7244.1240 10797032 PMC27365

[B36] World Health Organization Obesity and overweight fact sheet 2003 Geneva, Switzerland: World Health Organization Internet: http://www.who.int/dietphysicalactivity/media/en/gsfs_obesity.pdf (accessed 10 January 2013)

[B37] American College of Sports Medicine ACSM’s guidelines for exercise testing and prescription 2008 Philadelphia: Lippincott, Williams & Wilkins

[B38] Clausen JP Effect of physical training on cardiovascular adjustments to exercise in man Physiol Rev 1977 57 4 779 815 333481 10.1152/physrev.1977.57.4.779

[B39] Cornelissen VA Fagard RH Effects of endurance training on blood pressure, blood pressure-regulating mechanisms, and cardiovascular risk factors Hypertension 2005 46 4 667 675 10.1161/01.HYP.0000184225.05629.51 16157788

[B40] Rakobowchuk M Tanguay S Burgomaster KA Howarth KR Gibala MJ MacDonald MJ Sprint interval and traditional endurance training induce similar improvements in peripheral arterial stiffness and flow-mediated dilation in healthy humans Am J Physiol Regul Integr Comp Physiol 2008 295 1 R236 R242 10.1152/ajpregu.00069.2008 18434437 PMC2494806

[B41] Macpherson RE Hazell TJ Olver TD Paterson DH Lemon PW Run sprint interval training improves aerobic performance but not maximal cardiac output Med Sci Sports Exerc 2011 43 1 115 122 10.1249/MSS.0b013e3181e5eacd 20473222

[B42] Gibala MJ Little JP Van Essen M Wilkin GP Burgomaster KA Safdar A Raha S Tarnopolsky MA Short-term sprint interval versus traditional endurance training: similar initial adaptations in human skeletal muscle and exercise performance J Physiol 2006 575 Pt 3 901 911 16825308 10.1113/jphysiol.2006.112094 PMC1995688

[B43] Ortega FB Artero EG Ruiz JR Espana-Romero V Jimenez-Pavon D Vicente-Rodriguez G Moreno LA Manios Y Beghin L Ottevaere C Physical fitness levels among European adolescents: the HELENA study Br J Sports Med 2011 45 1 20 29 10.1136/bjsm.2009.062679 19700434

[B44] Bogdanis GC Nevill ME Boobis LH Lakomy HK Contribution of phosphocreatine and aerobic metabolism to energy supply during repeated sprint exercise J Appl Physiol 1996 80 3 876 884 8964751 10.1152/jappl.1996.80.3.876

[B45] Spriet LL Lindinger MI McKelvie RS Heigenhauser GJ Jones NL Muscle glycogenolysis and H+ concentration during maximal intermittent cycling J Appl Physiol 1989 66 1 8 13 2917960 10.1152/jappl.1989.66.1.8

[B46] Hazell TJ Macpherson RE Gravelle BM Lemon PW 10 or 30-s sprint interval training bouts enhance both aerobic and anaerobic performance Eur J Appl Physiol 2010 110 1 153 160 10.1007/s00421-010-1474-y 20424855

[B47] Baquet G Guinhouya C Dupont G Nourry C Berthoin S Effects of a short-term interval training program on physical fitness in prepubertal children J Strength Cond Res 2004 18 4 708 713 15574071 10.1519/13813.1

[B48] Durandt J Tee JC Prim SK Lambert MI Physical fitness components associated with performance in a multiple-sprint test Int J Sports Physiol Perform 2006 1 2 150 160 19114747 10.1123/ijspp.1.2.150

[B49] Lionis C Kafatos A Vlachonikolis J Vakaki M Tzortzi M Petraki A The effects of a health education intervention program among Cretan adolescents Prev Med 1991 20 6 685 699 10.1016/0091-7435(91)90064-B 1766941

[B50] Manios Y Kafatos A Health and nutrition education in elementary schools: changes in health knowledge, nutrient intakes and physical activity over a six year period Public Health Nutr 1999 2 3A 445 448 10610086 10.1017/s1368980099000610

[B51] Luepker RV Perry CL McKinlay SM Nader PR Parcel GS Stone EJ Webber LS Elder JP Feldman HA Johnson CC Outcomes of a field trial to improve children's dietary patterns and physical activity. The Child and Adolescent Trial for Cardiovascular Health. CATCH collaborative group JAMA 1996 275 10 768 776 10.1001/jama.1996.03530340032026 8598593

