# Supplementary material for: High intensity interval running enhances measures of physical fitness but not metabolic measures of cardiovascular disease risk in healthy adolescents
Source: BMC Public Health. 2013 May 24;13:498. doi: 10.1186/1471-2458-13-498 (PMC3666892; doi:10.1186/1471-2458-13-498)
Supplement: Additional file 1 — Focus Group Script – Participants. [file 1471-2458-13-498-S1.docx]

**Focus Group Script – Participants**

INTRODUCTIONS

Please take a moment to tell us something about yourself (Ice-breaker topic)

QUESTIONS

1. What are some of the reasons why you became involved with the project?

Probe: What did you hope to accomplish?

2. What did you like about the project?

Probe: What were some of the highlights or “best things” about the project?

3. What did you dislike about the project?

4. Was there any particular reason(s) why you continued with the project?

5. Do you think you will continue with the intervention now that the project has finished?

6. What have you learned from your experiences with the project?

7. Are there any other comments you would like to make?
