# Supplementary material for: High intensity interval running enhances measures of physical fitness but not metabolic measures of cardiovascular disease risk in healthy adolescents
Source: BMC Public Health. 2013 May 24;13:498. doi: 10.1186/1471-2458-13-498 (PMC3666892; doi:10.1186/1471-2458-13-498)
Supplement: Additional file 2 — Focus Group Script – PE Teachers. [file 1471-2458-13-498-S2.docx]

**Focus Group Script – PE Teachers**

INTRODUCTIONS

Please take a moment to tell us how long you have been a PE teacher for (Ice-breaker topic).

1. How did you find the project?

2. What were your expectations of the project?

3. What impact do you think the project had on the participants?

4. Why do you think the participants continued with the project?

5. Are there any other comments you would like to make?
